# Overview of Experimental and Clinical Findings regarding the Neuroprotective Effects of Cerebral Ischemic Postconditioning

**DOI:** 10.1155/2017/6891645

**Published:** 2017-04-04

**Authors:** Di Ma, Liangshu Feng, Fang Deng, Jia-Chun Feng

**Affiliations:** Department of Neurology and Neuroscience Center, the First Hospital of Jilin University, Changchun 130021, China

## Abstract

Research on attenuating the structural and functional deficits observed following ischemia-reperfusion has become increasingly focused on the therapeutic potential of ischemic postconditioning. In recent years, various methods and animal models of ischemic postconditioning have been utilized. The results of these numerous studies have indicated that the mechanisms underlying the neuroprotective effects of ischemic postconditioning may involve reductions in the generation of free radicals and inhibition of calcium overload, as well as the release of endogenous active substances, alterations in membrane channel function, and activation of protein kinases. Here we review the novel discovery, mechanism, key factors, and clinical application of ischemic postconditioning and discuss its implications for future research and problem of clinical practice.

## 1. Introduction

Ischemic preconditioning has been widely adopted as a clinical strategy aimed at protecting the brain from subsequent, more serious ischemia-reperfusion insults. Ischemic preconditioning involves the application of a brief, subthreshold episode of ischemia prior to the occurrence of irreversible ischemic injury [1]. A number of clinical trials have also confirmed that such preconditioning strategies attenuate the pathophysiological consequences of ischemia-reperfusion injury prior to cardiac bypass surgery [2, 3]. Based on the concept of ischemic preconditioning, research has begun to focus on the development of a nonpharmacological neuroprotective strategy that can be administered following the onset of ischemia. Most research regarding this strategy—termed ischemic postconditioning—has focused on the heart [4]. However, several proof-of-principle studies have yielded promising results for the brain as well [5–7].

## 2. Ischemic Postconditioning

Ischemic postconditioning was initially defined in the field of myocardial ischemia as a series of brief mechanical occlusions and reperfusions [8, 9]. Numerous studies have revealed that ischemic postconditioning exerts its effects via activation of endogenous neuroprotective mechanisms. In clinical research, inflation and deflation of the angioplasty balloon after reopening of the coronary artery can mimic repetitive coronary artery clamping during percutaneous transluminal coronary angioplasty (PTCA). This may be considered to be a type of ischemic postconditioning that has been proven effective for myocardial protection [10, 11]. In the central nervous system (CNS), ischemic postconditioning is often performed by mechanically blocking blood flow (such as that from the middle cerebral artery [MCA]) to the target region, or by using other methods, such as low doses of anesthesia, drugs with neuronal toxicity, or hypothermia.

Depending on the processing timeframe, ischemic postconditioning can be classified as either rapid ischemic postconditioning (RIPO) or delayed ischemic postconditioning (DIPO). RIPO is conducted within a few seconds to minutes following ischemia-reperfusion [12–15], whereas DIPO begins as early as a few hours or as late as 2 days following reperfusion [16, 17].

**Figure 1:**
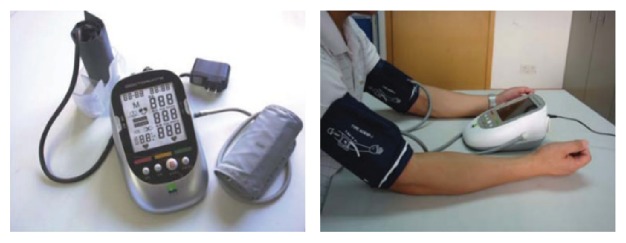
The BAIPC device patented by Xunming Ji from Xuanwu Hospital.

Ischemic postconditioning can also be defined according to the site of mechanical blockage as distal, proximal, or remote. Proximal ischemic postconditioning usually involves occlusion of the carotid artery, while distal ischemic postconditioning usually involves occlusion of the upper brachial artery. Remote ischemic postconditioning, on the other hand, involves occlusion of an artery in the lower limb. The bilateral arm ischemic preconditioning (BAIPC) device patented by Xunming Ji from Xuanwu Hospital can perform regular occlusion/reperfusion automatically and record the timing of every BAIPC process in real time. This device may be used in the RIPO clinical research (Figure 1).

The BAIPC device and how it is used are as follows. The device can perform 5 minutes of ischemia followed by 5 minutes of reperfusion automatically and record the timing of every BAIPC process in real time. It can also record heart rate and blood pressure. The time intervals for the BAIPC can be adjusted based on the requirements of the study.

## 3. The Protective Parameters of Ischemic Postconditioning

As in myocardial ischemia, the protective strength of cerebral ischemic postconditioning depends on temporal factors associated with the mechanical interruptions, such as the number of ischemia-reperfusion cycles and the durations of occlusion and reperfusion.

### 3.1. Therapeutic Window

The therapeutic window for the beneficial effects of ischemic postconditioning also depends on the type of technique used. The therapeutic window for RIPO is usually a few minutes to several hours after ischemia/reperfusion, while that for DIPO is usually from several hours to days after ischemia/reperfusion. Both RIPO and DIPO can effectively reduce cerebral infarction volume and improve recovery of neural function when initiated within the therapeutic window.

However, there is no uniform standard regarding the therapeutic window of either form of ischemic postconditioning. To date, research has been conducted using various models of ischemia in a number of different experimental animals and under various experimental conditions. Thismakes it difficult to adopt an appropriate standard from the available literature.

Variations in the results of a number of previous studies highlight the need to establish such a standard. For example, Jang et al. developed a rabbit model of spinal cord ischemia-reperfusion via occlusion of the infrarenal aorta and observed that ischemic postconditioning initiated either 1 minute or 5 minutes following ischemia-reperfusion was effective in improving neurological function and preserving motor neurons. However, no such neuroprotective effects were observed when ischemic postconditioning was initiated 10 minutes after reperfusion [18]. However, Gao et al. reported no change in infarct volume when ischemic postconditioning was initiated 3 minutes following reperfusion, though the technique employed in that study was rather different. Specifically, permanent middle cerebral artery occlusion was combined with 30 minutes of bilateral carotid artery occlusion to construct a rat model of ischemia. This was followed by immediate and repeated blocking and unblocking of the carotid artery as the postconditioning treatment [19]. Yet another study reported significantly reduced infarct volumes following focal cerebral ischemia and reperfusion of rat cerebral arteries when postconditioning was initiated within 2 minutes, 5 minutes, or 10 minutes after reperfusion. No such effects were observed when postconditioning was initiated 30 minutes after reperfusion [20].

Divergent results regarding the therapeutic window have been reported for DIPO as well. In a study by Burda et al., postconditioning performed by occlusion of the internal carotid artery 48 hours after ischemia-reperfusion in a rat model of cerebral ischemia resulted in significant improvements in the structure and function of nerve cells [21]. However, other researchers have reported that remote ischemic postconditioning involving occlusion of arteries in the hind limb results in significant neuroprotective effects when the treatment is initiated at 3 and 6 hours after reperfusion [22]. These results indicate that the effective therapeutic window for delayed postconditioning may range from a few hours to a few days.

Though there is no uniform standard to define the therapeutic window for ischemic postconditioning, the results of the aforementioned studies indicate that the therapeutic window may be modified by changing the site of occlusion, number of cycles, or additional factors, thus enhancing the clinical potential of such treatment.

### 3.2. Ischemia-Reperfusion Cycles

Ischemic postconditioning refers to the process of inducing a series of brief periods of ischemia and reperfusion following lethal ischemic injury to a specific organ in order to reduce the overall extent of ischemic injury. The event consisting of one ischemic period and one subsequent reperfusion period is defined as one cycle. In most animal models, three to ten cycles of postconditioning are performed, though some clinical research studies have utilized between three and five cycles.

In a 2008 study by Gao et al., focal ischemia was generated by permanent occlusion of the left distal middle cerebral artery (dMCA) combined with 30 minutes of occlusion of both common carotid arteries (CCAs) in rats [22]. Postconditioning involved brief repetitive release and occlusion of the CCAs after 30 minutes of continuous occlusion. Three cycles—but not 10 cycles—of postconditioning consisting of 10 seconds of CCA occlusion followed by 30 seconds of CCA release (30 seconds/10 seconds) significantly reduced infarct volume measured 2 days after stroke. However, postconditioning with 10 cycles—but not with three cycles—of 10-second CCA occlusion/10-second CCA release significantly reduced infarct volume, although no such neuroprotective effects were observed when postconditioning was initiated 3 minutes after reperfusion. The results of the aforementioned studies indicate that the number of postconditioning cycles influences the ability of the treatment to induce neuroprotective effects [23, 24]. This notion stands in contrast to results observed in myocardial ischemic postconditioning experiments, where factors such as therapeutic window and loop of ischemia-reperfusion time, rather than cycle number, determine the efficacy of the treatment [25, 26]. This may be due to differences in the sensitivity of heart and nerve cells to energy loss and to the absence of a penumbra in myocardial ischemia.

### 3.3. Duration of Reperfusion and Occlusion

Rezazadeh et al. developed a rat model of embolic stroke by embolizing a preformed clot into the MCA. This was followed by ischemic postconditioning involving blockage and release of the bilateral CCAs [27]. Postconditioning was performed after 30 minutes of MCA occlusion (MCAO). Rezazadeh et al. observed that postconditioning with five cycles of 10-second occlusion and 30-second reperfusion of the bilateral CCAs reduces ischemic damage and neurological deficits induced by embolic stroke in rats, while occlusion and release times of 30 or 60 seconds resulted in no such protective effects. As previously mentioned, Gao et al. [22] reported that three cycles of 30 seconds/10 seconds effectively reduce infarct volume and induce neuroprotective effects following 30 minutes of CCA occlusion, although some research suggests that three cycles of 10 seconds/10 seconds may also reduce infarct volume without offering neuroprotective benefits [27]. The results of these experiments indicate that the durations of reperfusion and occlusion are also key factors influencing the effects of ischemic postconditioning.

### 3.4. Proper Time for Observation

The proper time for the observation of the effects is another important factor when assessing the protective effects of ischemic postconditioning when determining whether the hypothetical neuroprotective effects have been achieved. The effects of postconditioning on some clinical indicators such as cerebral blood flow and infarct volume can usually be observed 2-3 days after ischemic stroke.

While research regarding RIPO is abundant, the study of DIPO has been relatively limited to date. Most previous studies have focused only on whether neuroprotective effects have occurred and have therefore utilized observation times of 2-3 days following stroke. Based on the onset time and the conditions of clinical emergencies, there is no doubt that DIPO has much higher potential for clinical application than RIPO. Most importantly, future research should stress the use of an appropriate therapy window to guide clinical practice. We could not make the conclusion that DIPO had no side effects when compared to RIPO. The mechanism of neuroprotection of DIPO, especially in the central nervous system, is another focus of study, as this effect is transferred from peripheral tissue to the central nervous system.

In summary, although it is possible to alter a number of factors involved in the control of ischemic postconditioning in the brain, it remains difficult to determine the appropriate solution for each individual. Due to the complexity of the clinical environment, it may be possible to adjust some factors, such as the postconditioning method (proximal or distal) or the number of cycles, in order to determine the ideal therapeutic schedule. Therefore, further studies regarding the mechanisms underlying the neuroprotective effects of and factors influencing ischemic postconditioning remain particularly important.

## 4. Ischemic Preconditioning and Ischemic Postconditioning

### 4.1. The Idea of Ischemic Postconditioning Originated from That of Ischemic Preconditioning

**Figure 2:**
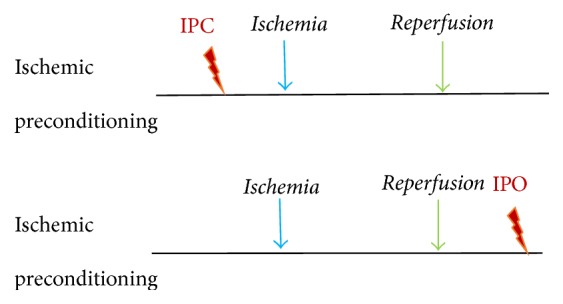
*Ischemic preconditioning (IPC) and ischemic postconditioning (IPO)*. IPC is performed prior to ischemia, while IPO is performed following ischemia-reperfusion.

Preconditioning refers to the process of inducing brief periods of subthreshold ischemia in order to prevent or attenuate severe ischemic injury due to subsequent, prolonged periods of ischemia [28, 29]. In light of the neuroprotective effects of preconditioning on cerebral ischemia, researchers began to consider the potential benefits of ischemic postconditioning. Following confirmation of the protective effects of ischemic postconditioning on myocardial ischemia in animals, researchers observed similar success in translating these effects to humans [30, 31]. Indeed, the protective effects of ischemic postconditioning have been observed not only in the heart, but also in the vasculature and various other parenchymal organs. Ischemic postconditioning has thus often resulted in improved clinical outcomes and prognosis [32]. Translating pre- and postconditioning into clinical settings will require the combination of basic science research and clinical testing (Figure 2).

### 4.2. Mechanisms of Preconditioning and Postconditioning

Despite the fact that ischemic preconditioning and postconditioning are applied along distinctly different time courses, both share several common protective mechanisms involving modification of key mitochondrial targets or activation of reperfusion injury salvage kinase (RISK) pathways. These may involve the Akt, extracellular signal-regulated kinase 1/2 (ERK1/2), and mitogen-activated protein kinase (MAPK) pathways [33–35]. The neuroprotective effects of both ischemic preconditioning and ischemic postconditioning may involve the activation of C-C chemokine receptor type 2 [36]. A significant role of the pannexin 1 (Panx1)/P2X7 receptor complex in the cardioprotective mechanisms of ischemic preconditioning and postconditioning (IPC) has been established. We investigated whether Panx1/P2X7 purinoceptors are also involved in the neuroprotective mechanisms of IPC in mice [37]. Furthermore, the degree of neuroprotection observed following postconditioning is equivalent to that observed in models of ischemic preconditioning [4].

The acute protective effects of IPC likely result from immediate posttranslational protein modifications (e.g., phosphorylation) within cell energetic or survival systems. In contrast, the protective effects of IPC likely result from protein synthesis of previously dormant genes involved in angiogenesis, energy metabolism, vasomotor control, inflammation, and cell survival (e.g., growth factors). Therefore, elucidation of the cell signaling pathways underlying the protective effects of ischemic preconditioning may provide insight into those underlying the effects of ischemic postconditioning.

### 4.3. Are Synergistic Effects Observed When Preconditioning and Postconditioning Are Combined? 

Gao et al. investigated the effects of combining preconditioning with postconditioning treatment on ischemic damage [22]. Rapid preconditioning involved transient occlusion of the left dMCA for 15 minutes using an aneurysm clip, followed by permanent dMCA occlusion and prolonged (30 minutes) CCA occlusion. Rapid preconditioning was combined with 10 cycles of 10-second/10-second postconditioning conducted immediately after reperfusion. Gao et al. also investigated delayed preconditioning by inducing occlusion of the left dMCA for 5 or 15 minutes three days prior to the induction of prolonged ischemia. This was followed by 10 cycles of 10-second/10-second postconditioning immediately after reperfusion. The authors observed that the combination of rapid preconditioning with 15 minutes of dMCA occlusion and postconditioning consisting of three 30-second/10-second cycles did not further reduce infarction when compared with the individual treatments. We also did not observe any further reduction in infarct volume with a combination of delayed preconditioning with 15 minutes of dMCA occlusion and the same postconditioning. However, a number of experimental and clinical studies have strongly indicated that some combination of ischemic preconditioning and postconditioning may offer enhanced neuroprotective effects, whereas others are in disagreement [38, 39].

## 5. Neuroprotective Effects of Ischemic Postconditioning

### 5.1. Minimization of Damage in the Ischemic Penumbra

Minimizing damage in the ischemic penumbra, which requires the attenuation/prevention of neural cell apoptosis, is the current primary therapeutic target in the treatment of acute stroke. Research indicates that remote ischemic postconditioning may protect against ischemic damage in the brain via the p38 MAPK signaling pathway, improve neuronal morphological changes in the area of the ischemic penumbra, and reduce neuronal cell apoptosis in rat models of focal cerebral ischemia/reperfusion (I/R) [40]. Furthermore, a recent study suggests that inhibition of autophagic pathways plays a key role in IPC-induced neuroprotection against focal cerebral ischemia [41].

Recent studies have reported markedly increased autophagy following the upregulation of LC3/Beclin 1 and downregulation of p62 in the penumbra at various time intervals following ischemia. Furthermore, ischemic postconditioning performed at the onset of reperfusion reduces infarct size, mitigates brain edema, inhibits the induction of LC3/Beclin 1, and reverses decreases in p62 [42].

Other researchers have reported that the protective effects of remote limb ischemic postconditioning against cerebral I/R injury may be related to the attenuation of neuronal apoptosis and inflammation via activation of signal transducer and activator of transcription 3 (STAT3), as well as attenuation of tumor necrosis factor-*α* (TNF-*α*) and nuclear factor-*κ*B (NF-*κ*B) protein expression in the ischemic penumbra. Additional studies have suggested that ischemic postconditioning protects against focal cerebral ischemia by inhibiting brain inflammation while attenuating peripheral lymphopenia in mice [43].

### 5.2. Contribution to Cerebral Collateral Circulation

**Figure 3:**
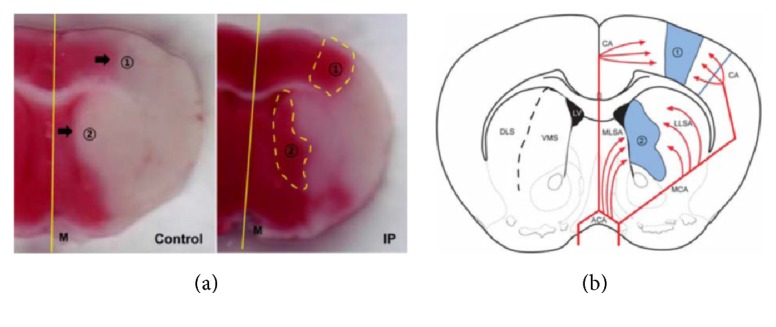
*Effect of ischemic postconditioning on collateral circulation*. Ischemic postconditioning can effectively reduce the infarct area as well as the infarct border zones between the cortical territories of the ACA and those of the MCA (lenticulostriate artery supply area) in a rat model of MCAO. (a) The spared regions of the infarct area following ischemic postconditioning are observed in border zones between the cortical territories of the ACA and those of the MCA (area 1), as well as in the ventromedial and dorsolateral striatum (area 2). (b) The schematic diagram shows brain regions with collateral blood supply. The cerebral collateral circulation may be defined as a subsidiary vascular network that is dynamically recruited after arterial occlusion and represents a powerful determinant of ischemic stroke outcome. The red lines signify blood supplied by cerebral arteries, and the blue regions in the schematic drawing indicate the “fighting area” between collaterals of the ACA and MCA, as well as the medial lenticulostriate artery (MLSA) of the ACA and the lateral lenticulostriate artery (LLSA) of the MCA. CA; cortical artery, DLS; dorsolateral striatum, M; midline, VMS; ventromedial striatum; ACA: anterior cerebral artery; MCA: middle cerebral artery; MCAO: middle cerebral artery occlusion.

In a study by Joo et al., ischemic postconditioning consisted of a series of brief occlusions of the MCA after reperfusion in a mouse model of focal ischemia. As a result, spared infarct areas were observed in the border zones between the cortical territories of the ACA and MCA, as well as in the ventromedial and dorsolateral striatum. These regions have been confirmed to be affected by ischemia sequentially over longer periods following onset of ischemia in the dorsolateral striatum. Ischemia then progresses into the ventromedial striatum and the cerebral cortex in the MCA territory (Figure 3) [43]. The ischemic regions spared by ischemic postconditioning can thus be regarded as the ischemic penumbra. Results from additional studies suggest that the therapeutic effects of postconditioning may involve the promotion of neurogenesis and angiogenic remodeling during the recovery phase after focal cerebral ischemia via an increase in the numbers of doublecortin/BrdU and collagen-IV/Ki67-positive cells [44].

RIPO acts at the cellular level to directly protect the vascular endothelium via KATP channel-dependent mechanisms [24]. Vascular endothelial dysfunction triggered by ischemia/reperfusion can promote vasoconstriction and thrombosis thorough loss of endothelium-derived factors, such as nitric oxide (NO), while postconditioning has been shown to preserve endothelial function, increase NO production, and decrease adhesion of neutrophils to endothelial cells [45].

### 5.3. Increases in Cerebral Blood Flow

Disturbances in cerebral blood flow (CBF) occur throughout the period of reperfusion following ischemic stroke. In fact, it has been reported that the clinical neuroprotective effects of remote ischemic conditioning (RIC) are partially related to improvements in CBF. Following reperfusion, there is a short period of hyperperfusion, followed by a longer period of hypoperfusion. A recent study indicates that combined ischemic postconditioning may stabilize CBF disturbances during the early hyperperfusion and later hypoperfusion periods [20]. Furthermore, Liu et al. have reported that three 30-second/30-second cycles of postconditioning are optimal for improving CBF, increasing NO synthesis, and reducing brain injury following cerebral ischemia [46]. The authors concluded that NO is a reliable candidate in mediating the neuroprotective effects of ischemic postconditioning. Nitrite is a key circulating mediator of RIC and may be a mediator of increased CBF and cytoprotection via its effects on nitrosylation of mitochondrial proteins, such as complex I [47].

### 5.4. Reduced Damage to the Blood-Brain Barrier and Attenuation of Brain Edema

Disruptions in the energy and material supply of brain tissue during cerebral ischemia, accompanied by the failure of ion pumps in the cell membrane, lead to cerebral edema. Numerous studies have reported that limb remote ischemic postconditioning (LRIP) significantly reduces cerebral infarct volume and relieves brain edema. Possible mechanisms underlying the protective effects of LRIP may include amelioration of endothelial dysfunction, maintenance of the integrity of the blood brain barrier, modulation of protein synthesis and nerve activity [48–50], inhibition of apoptosis [40], and decreases in reactive oxygen species (ROS) [51].

In remote postconditioning of cerebral ischemia in rats, downregulation of aquaporin 4 (AQP4), which is involved in water homeostasis in astrocytes, may attenuate cerebral damage after transient MCAO [52]. Previous studies indicate that IPC may significantly improve neurological function, decrease infarct volume and edema, and increase the integrity of the blood-brain barrier (BBB) [53]. Yu et al. have reported that ischemic postconditioning exerts neuroprotective effects in neonatal rats with hypoxic-ischemic brain damage (HIBD) and may relieve cerebral edema by regulating the expression of AQP4 [54].

### 5.5. Improvements in Cognitive Function

Chronic cerebral ischemia leads to cognitive dysfunction, although similar neuronal damage and dysfunction are also observed in vascular dementia, Alzheimer's disease, and Binswanger's disease [55]. Ischemic postconditioning has been reported to attenuate brain damage caused by chronic cerebral ischemia and may also improve cognitive and neural function following such insults. Experimental evidence suggests that delayed ischemic postconditioning slows the process of ischemic injury with regard to hippocampal cone deformation [56, 57] and may also increase endothelial nitric oxide synthase (eNOS) and Src kinase levels in order to protect nerve cells in the hippocampus.

The highest density of N-methyl-D-aspartate (NMDA) receptors is found in the hippocampal CA1 and CA3 areas and the dentate gyrus, which are areas closely associated with cognitive function. Ischemic postconditioning activates NMDA2A receptors, promotes the internal flow of calcium ions, influences the ERK pathway and the synthesis of NO, and restores hippocampal blood flow [58]. Ischemic postconditioning can also inhibit some types of NMDA receptors, such as kainate receptors, in order to reduce glutamine toxicity and promote the recovery of cognitive function [21]. Cerebral ischemic postconditioning also influences KCC2 pathways and regulates the expression of gamma-aminobutyric acid receptors, resulting in improvements in cognitive dysfunction following stroke [59].

## 6. Signaling Molecules and Mechanisms in Conditioning

To date, hundreds of studies have reported the involvement of different signaling molecules and potential mechanisms underlying the effects of postconditioning under a wide range of experimental conditions. Studies have demonstrated the effects of ischemic postconditioning on activation of adenosine, bradykinin (BK), and endogenous protective molecules such as NO and G-protein-mediated kinases, which further act on the mitochondria, endoplasmic reticulum, or nucleus and produce neuroprotective effects in targeted tissues [60–62].

### 6.1. Protective Effects on Vascular Endothelial Cells

In animal models of brain ischemia, rapid ischemic postconditioning can be triggered by promoting the synthesis of eNOS and activating the PI3K/Akt signal transduction pathway, which act to protect vascular endothelial cells and promote vascular remodeling [51]. Pignataro et al. have reported that remote postconditioning promotes phosphorylation of the ERK signaling pathway, accelerates neuronal NOS synthesis, and protects blood vessels from damage [63]. In a study on vascular dementia, Khan et al. reported that ischemic postconditioning inhibits expression of the inflammatory factor TNF-*α*, reduces expression of intercellular adhesion molecules (ICAMs), protects vascular endothelial cells, and inhibits inflammatory factor aggregation and infiltration, resulting in protective effects on blood vessels [64]. Endothelial cells are important components of the blood brain barrier, and research has indicated that postconditioning increases expression of occludin, blocking the infiltration of harmful factors and protecting nerve vascular cells by maintaining the integrity of the blood-brain barrier [53].

### 6.2. Reductions in Oxidative Damage

As is the case in myocardial ischemic reperfusion injury, while free radicals may be generated to a small extent during ischemia, far greater production of reactive oxygen intermediates occurs after reintroduction of oxygen during cerebral ischemic reperfusion. Most of the protective mechanisms of ischemic postconditioning are the same in the heart and the brain, although the effects of postconditioning on ROS are controversial.

In myocardial ischemic reperfusion injury, ROS signaling is an essential trigger of ischemic and pharmacological postconditioning. Chemically blocking the production of ROS abolishes the protective effect of ischemic postconditioning in the heart [65, 66]. On the other hand, in cerebral ischemic postconditioning, ROS play a harmful role that postconditioning should overcome. Elimination of ROS during postconditioning may involve reducing the number and activation of neutrophils in the rat brain and peripheral blood following LRIP. This may be linked to the downregulation of NADPH oxidase activity in neutrophils by the MyD88/TRAF6/p38-MAPK pathway. In fact, there is abundant evidence that, under IRI, activated neutrophils are considered to be the main source of ROS [67–69].

**Figure 4:**
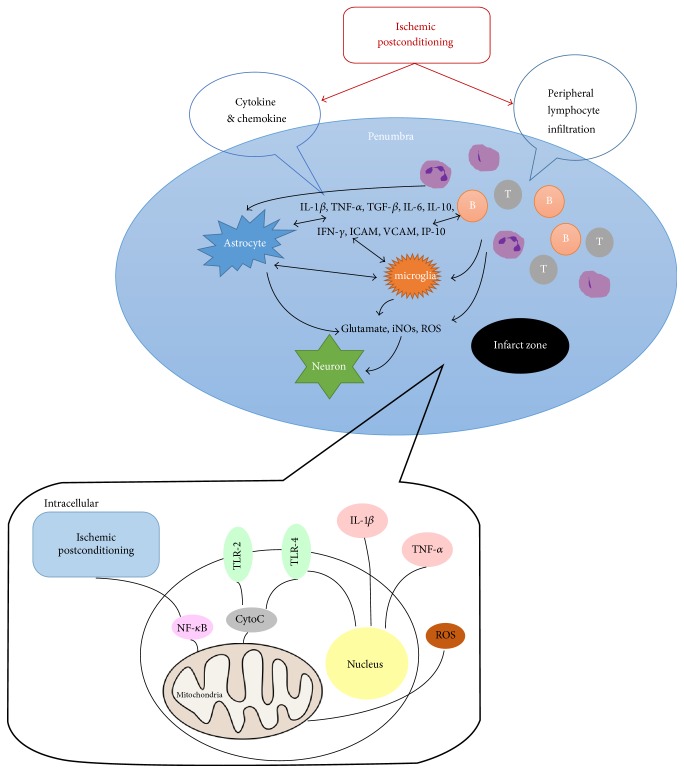
*Mechanism of the anti-inflammatory effects of ischemic postconditioning (IPO)*. Ischemic postconditioning places stress on the cell, triggering endogenous protective mechanisms. Immunosuppression is reduced by decreasing peripheral humoral immunity.* Intracellular*: ischemic postconditioning places stress on the cell, triggering endogenous protective mechanisms. Reduction of mitochondrial cytochrome C results in immunosuppression, which leads to a decrease in the levels of inflammatory cytokines and chemokines. IL-1*β*: interleukin 1 beta; TNF-*α*: tumor necrosis factor alpha; TLR-2: toll-like receptor 2; TLR-4: toll-like receptor 4; Cyto C: cytochrome C; NF-*κ*B: nuclear factor kappa-light-chain-enhancer of activated B cells.

Another research also indicates that ischemic postconditioning exerts its neuroprotective effects via ROS suppression. A study involving rat models of local cerebral ischemia indicates that rapid initiation of ischemic postconditioning within 30 minutes of reperfusion reduces the levels of peroxides and lipid peroxides, in turn reducing free radical damage [7, 70]. Furthermore, ischemic postconditioning has been shown to increase acetylcholine and NO synthesis and inhibit oxidative stress, thereby improving cognitive function (Figure 4) [71].

### 6.3. The Inflammatory Response following Ischemic Stroke

Xing et al. demonstrated that a decrease in the content of glutathione (GSH) together with an increase in myeloperoxidase (MPO) and proinflammatory markers may be observed in rats subjected to global cerebral ischemia/reperfusion [72]. A study by Kong et al. further revealed that rapid postconditioning can inhibit MPO activity and IL-1*β*, TNF*α*, and ICAM-1 expression while preventing leukocyte aggregation in the cerebral cortex (Figure 4) [73]. These results indicate that cerebral ischemic postconditioning may inhibit the invasion of inflammatory agents and further block secretion of proinflammatory cytokines and chemokines. Indeed, a study by Liang et al. revealed that IL-1*β* and IL-6 are reduced in both proximal and remote postconditioning [74].

Decreases in transient focal ischemia-induced infarct volume and rates of apoptosis have also been observed when ischemic postconditioning is induced within 24 hours of reperfusion following 2 hours of focal cerebral ischemia (Figure 4) [75]. Research has also indicated that ischemic postconditioning markedly attenuates reductions in NF-*κ*B/p65 in the cytoplasm and elevates its content in the nucleus 6 hours and 24 hours following reperfusion. Moreover, decreases in I*κ*B*α* and increases in phosphorylated I*κ*B*α* and phosphorylated NF-*κ*B/p65 are reversed by ischemic postconditioning.

It is known that T cells infiltrate areas of focal ischemia following stroke and that ischemic postconditioning effectively reduces the infiltration of T cells and total infarct volume (Figure 4) [76]. In a study of stroke-induced immunodepression, Joo et al. observed that ischemic postconditioning reverses reductions in immune cell numbers in the peripheral blood supply and improves systemic immunodepression (Figure 4) [43].

### 6.4. Antiapoptotic Effects

Rapid ischemic postconditioning significantly reduces the number of terminal deoxynucleotidyl transferase dUTP nick end labeled cells in the ischemic area when observed two days after stroke [7]. Mitochondrial ATP-sensitive potassium channels (mitoKATP) play key roles in mediating the protective effects induced by ischemic postconditioning. The mitochondrial proteins Bax and p53, as well as the antiapoptotic proteins Bcl-2 and Bcl XL, are also involved in ischemia-reperfusion-induced apoptosis. However, rapid postconditioning results in the release of cytochrome C from mitochondria in the cytoplasm, blocking cell apoptosis. Ischemic postconditioning may inhibit apoptosis by activating the TOPK (T-LAK cell-originated protein kinase/protein kinase B) pathway while promoting Akt phosphorylation in order to protect nerve cells and reduce infarct volume [77].

MAPK signaling pathways, including the ERK1/2, p38 lightning, and c-Jun amino terminal kinase (JNK) pathways, are closely related to the extent of ischemic injury and neuronal survival [78]. A study by Liu et al. revealed that, 1–24 hours following stroke, phosphorylation of ERK1/2 continues to increase, although rapid ischemic postconditioning significantly inhibits the expression of ERK1/2 in the ischemic penumbra [79]. Ischemic postconditioning also relieves NMDA2A receptor intracellular calcium overload, reduces the phosphorylation and expression of ERK, and promotes the expression of Bcl-2. These results indicate that phosphorylation of the ERK signaling pathway plays a key role in mediating the protective effects of rapid ischemic postconditioning [63].

Endoplasmic reticulum (ER) stress in ischemia-reperfusion injury is one of the most important factors that lead to cell apoptosis. Following ischemic postconditioning, the ER stress response results in elevated levels of C/EBP homologous protein (CHOP). This affects the release of Bim and Bcl-2, which interfere with the cell apoptosis pathway. Ischemic postconditioning can also cause rapid increases in GRP78 expression, dephosphorylation of EIF2*α*, decreases in caspase 12 and Bim expression, and increases in Bcl-2 expression, which all act to inhibit cell apoptosis [80, 81]. Ischemic postconditioning also downregulates cytochrome C release to the cytosol, Bax translocation to the mitochondria, and caspase 3 activity [82]. The results of the aforementioned studies indicate that ischemic postconditioning may reduce ischemic injury by blocking cell apoptosis.

### 6.5. Neuroprotective Protein Kinase Cell Signaling Transduction Pathways

**Figure 5:**
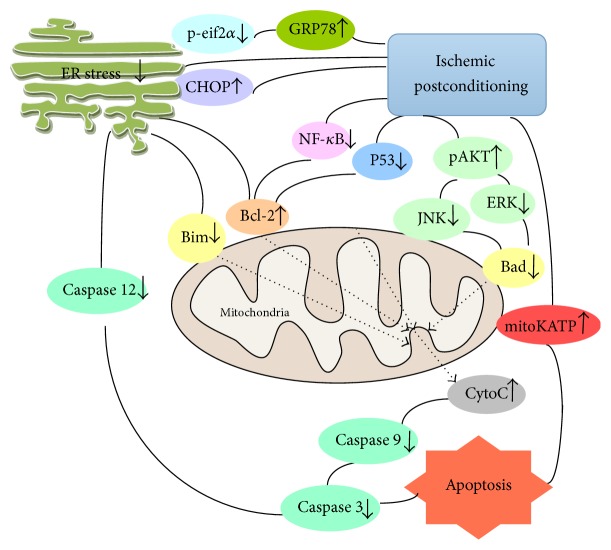
*Mechanism of antiapoptotic effects of ischemic postconditioning (IPO).* ER stress is closely related to the function of mitochondria. Ischemic postconditioning places stress on the cell, triggering endogenous protective mechanisms that reduce ER stress and protect the mitochondria. This attenuates apoptosis in the CNS. P-eif2*α*: phosphoeukaryotic initiation factor 2 alpha; GRP78: glucose-regulated protein 78; CHOP: C/EBP homologous protein; ER: endoplasmic reticulum; Bim: Bcl-2-like protein; Bcl-2: B-cell lymphoma 2; NF-*κ*B: nuclear factor kappa-light-chain-enhancer of activated B cells; JNK: c-Jun N-terminal kinase; pAKT: phosphorylated protein kinase B; ERK: extracellular signal-related kinase; Bad: Bcl-2-associated death promoter; mitokATP: mitochondrial ATP-sensitive potassium channels; Cyto C: cytochrome C.

Both ischemic preconditioning and postconditioning promote Akt phosphorylation and have neuroprotective effects. Research indicates that both rapid and delayed postconditioning influence important targets for neuroprotection (Figure 5) [7, 78, 79]. The prosurvival protein kinases ERK, p38 MAPK, and Akt have prolonged phosphorylation in the cortex of postconditioned rats (Figure 5) [63].

Phosphorylated Akt can also raise levels of mammalian target of rapamycin (mTOR) in order to promote neuroprotection. Ischemic postconditioning may result in time-dependent regulation of adenosine monophosphate-activated protein kinase (AMPK) activation and autophagy, and AMPK may strengthen the autophagy effect by inhibiting mTOR [83]. Indeed, some researchers have reported that the AKT/mTOR pathway plays a key role in the long-term protective effects of ischemic postconditioning [84].

Akt may indirectly participate in the inhibition of the mitochondrial apoptosis pathway in order to ensure the survival of cells following ischemic injury by influencing the activity of Bim and the phosphorylation of PKC, thereby affecting mitochondrial ATP-dependent potassium channels. Therefore, Akt signaling pathways may play a vital role in mediating the protective effects of ischemic postconditioning.

Postconditioning leads to increased Hsp70 expression and decreased NF-*κ*B and proteasome activities. Reduced infarct volume and proteasome inhibition were reversed by Hsp70 knockdown, suggesting a critical role of the Hsp70 proteasome pathway in ischemic postconditioning [84].

RIPO significantly upregulates the expression of nuclear factor erythroid 2-related factor 2, heme oxygenase-1, and quinone oxidoreductase-1 and the activity of superoxide dismutase, while downregulating the formation of malondialdehyde.

## 7. Clinical Translation

The standards for robust data on neuroprotective signaling have risen, and experiments utilizing single-dose antagonists are no longer satisfactory for the identification of steps within a signaling pathway. Unequivocal identification of a signaling step requires not only an appropriate conditioning protocol with infarct size (IS) as an endpoint, but also biochemical or immunoblotting data for signal activation. In fact, IS reduction following genetic ablation or pharmacological inhibition of the signal molecule is now routinely required.

To improve translation of experimental findings into clinically applicable standards, further insight into the mechanisms underlying postconditioning phenomena is required, although equal emphasis should be placed on the identification of novel signaling elements in potentially reductionist experimental models and on the translation of such novel, yet reductionist, findings into more complex and integrative models. In addition, future studies should focus on the identification of signaling elements involved in neuroprotection in the human CNS. Moreover, they should retrospectively evaluate experimental models that may have predicted these elements, develop standards for the identification of robust signaling elements that may serve as potential drug targets, and organize interactions between basic and clinical scientists in order to develop proof-of-concept clinical trials and to eventually carry out larger prospective multicenter trials.
